# Chicago Medical Society

**Published:** 1889-01

**Authors:** 


### SOCIETY REPORTS.

### *CHICAGO MEDICAL SOCIETY.*

*Regular Meeting, October 6.*

The President, J. H. Etheridge, M.D., in the Chair.

Dr. Bayard Holmes reported a case of Gastronomy, which appeared in The Journal and Examiner.

Dr. James G. Kiernan read a paper entitled

MENTAL SYMPTOMS AFTER SURGICAL PROCEEDINGS.

Under the title "delirium traumaticum nervosum" the older surgeons described a number of mental symptoms which made their appearance after surgical operations. These symptoms usually consisted of great motor agitation, visual and auditory hallucinations, excessive loquacity, and exceptionally the delirium was of a stuporous type, and still more rarely, the symptoms were of a very violent type. The patient generally made a rapid and full recovery. Exceptionally death resulted from the delirium, and still more exceptionally the patient remained permanently insane.

Scheede reported many cases due, he claimed, to iodoform; others were charged to carbolic acid, and still others to mercury bichloride. There was no great improbability in this etiology, for the mental phenomena were such as these agents do produce. Cases occurred, however, where the influence of antiseptics could be excluded. Then the symptoms were charged to blood poisoning, but in the majority of instances there was no evidence of this. This complication has been observed in 80 cases after various surgical procedures, according to a not very thorough research I have made of the literature. Thirty cases occurred after cataract operations. Thirty resulted after gynæcological procedures. The remainder resulted from plastic operations, amputations, dental procedures, etc. In 15 cases there was larvated epilepsy precedent to the operation. In 10 hysteria major was present. In 15 cases a neurotic element existed. In 25 cases the patients had arrived at periods of involution when instability of the nervous system usually results. In 6 cases the influence of blood poisoning was demonstrated.

The prognosis in the larvated epileptic cases was good so far as the mental phenomena was concerned. The same is true of the neurotic and hysterical cases. In some of the cases occurring at the period of involution senile degenerations were precipitated. The majority of the blood poisoning cases died. As a rule the type presented was that of acute confusional insanity. Ordinary epileptic and hysterical insanity resulted in cases of larvated epilepsy, and hysterical insanity in hysterics. Typhomania at times occurred.

According to certain estimates, about 2 per cent. of the gynæcological procedures and the same per cent. of cataract operations are followed by psychical symptoms. The etiology is clear in most cases. The antecedent conditions of the patient as to age and constitution act as a predisposing cause. The mental agitation precedent to the operation is a direct exciting cause.

The moral treatment of the patient antecedent to operation, supplemented by proper medicinal treatment, is the best prophylaxis. Sedatives and other measures tending to calm agitation are necessary. In most of these cases conium is indicated. Opium, and not chloral hydrate or the bromides, will, as a rule, be found of most value in a majority of cases. In hysterical and epileptic cases these latter remedies are indicated. In many of the hysterical cases salix nigra and monobromate of camphor give good results.

Dr. William Axford: I have observed this condition once or twice in old people. Last March I did a supra-pubic cystotomy and removed an enlargement of the prostate from an old gentleman of 79. For four days after the operation he was in a peculiar condition of delirium; there was no rise of temperature, no chill, nothing like sepsis, in point of fact it was an active delirium. The old gentleman was very smart; and he afterwards told me he had lost those four days entirely. I have always thought the condition was due to the influence of anæsthetics on the brain, but Dr. Kiernan informs me that the older physicians observed this phenomenon before anæsthetics were used.

Dr. J. H. Etheridge: During the month of June last, I took care of a woman in confinement; in the last of it ether was administered for three-quarters of an hour and delivery was effected with the forceps. The labor was particularly severe in every way and considerable laceration took place. After that etherization, she was delirious, and was not at all herself afterwards. She slept when night came, but throughout the whole of the time after that, while she lived, there was a sort of stilted, exalted condition of politeness about her that was entirely foreign to her. She ran along through the three days, so far as her puerperal condition was concerned, without a symptom arising, and on the fourth day she died, apparently from heart failure. I suppose this case would come under this head.

Dr. James G. Kiernan: With regard to Dr. Axford's case, I remember on one occasion seeing a very similar condition result from the passage of a catheter in an old man who had some prostatic difficulty. The catheter was passed by a surgeon of considerable skill who became considerably alarmed at the denouement. The man passed into a state of exaltation and was difficult to manage; he insisted on going out and attempting to run a race. There is very little doubt that old men about the period of sixty are very easily upset by operations of this kind.

Dr. Arthur R. Reynolds exhibited an

ACEPHALUS FŒTUS, given birth to by a primipara, 21 years of age, at full term of utero-gestation. When called to the patient, I found she had been suffering moderate labor pains for about 24 hours, the abdomen was greatly distended, the os was fully dilated, and the membranes were well down in the vagina. I ruptured the membranes, passed my finger through the opening and found the fœtus floating freely in the amniotic fluid. The character of the presenting part at once arrested my attention. I was quite undecided whether I had a head or a breach presenting, but was inclined to think it was the breach. While pressing the examination, the fœtus made a violent and startling convulsive movement, and afterward continued to do so every time I touched the presenting part, the cause of which I did not recognize until after the labor was completed. The quantity of amniotic fluid was very large. It flooded the bed and ran down on the floor. After the water had escaped the uterine contractions became strong and expulsion was completed in half an hour in the second position. Notwithstanding the activity of the fœtal movements I found, to my astonishment, that the child was still-born. The cord was small and pulseless and there were none of the ordinary signs of asphyxia.

The fœtus measures fourteen inches in length and weighs three and a half pounds. The rudimentary and brainless head rests immediately upon the shoulders, there being no neck whatever. The cranium and spinal cord are open as far down as the second or third dorsal vertebra, a condition that is characterized by the term deranencephalia. The fœtus is plump and well developed with broad shoulders and well rounded trunk and limbs, and it is of the female sex, all of which is the rule with this class of monstrosities.

The convulsive movements that startled me so in the beginning of the second stage, were evidently produced by my finger pressing directly on the stump of the brain and eliciting one of the signs of the acephalic fœtus. It was by this sign that Cazeaux was enabled to diagnose a fœtus of this kind before the membranes were ruptured, to the astonishment of the students at the clinic where the labor occurred. That the child should be born dead, when it was certainly alive half an hour before, excites a query as to the cause. Although my manipulations were by no means severe, I incline to the belief that death was caused by the pressure of the fingers upon the nerve centers.

Dr. S. V. Clevenger: I remember ten years ago Dr. Roler described an anencephalic monster which he had delivered. He said he believed during delivery that from the rebound every time his finger touched the os, he had a monster of that kind in utero, because he had been just reading an article, in the *American Journal of Obstetrics*, of a similar instance. It is an important diagnostic indication.

Dr. S. V. Clevenger read a paper entitled: *The Origin of Sexual Perversions.*

Dr. J. G. Kiernan read a paper entitled: *Sexual Perversion as Illustrated by the Whitechapel Murders.*

Dr. Arthur R. Reynolds, reported

A CASE OF SEXUAL PERVERSION—

that of a man who could have sexual relations only with women who had lost the right leg.

Dr. J. W. Nelson reported

A CASE OF SPONTANEOUS PYÆMIA.

I have entitled this paper "A Case of Spontaneous Pyæmia." The title should, perhaps, be followed by an interrogation point: *First:* Because some writers doubt that pyæmia can originate without a previous lesion of some kind: and, *Second:* Being unable to hold an autopsy (for the patient died), I was unable to confirm my diagnosis by finding multiple abscesses, or other signs of embolism that are usually present. I think, however, that the case was undoubtedly one of pyæmia, and if there is such a thing as spontaneous pyæmia, this would come under that head, for there was no history or sign of wound, bruise or injury of any kind. I submit the case not merely to put it on record, but to emphasize the necessity of care in diagnosticating between such cases and acute articular rheumatism. For I was obliged to make a second diagnosis, and that is an unpleasant thing to do when your patient is in *articulo mortis.*

On August 8, 1888, I was called to see a boy seven years old, August P.; parents illiterate Germans; sanitary surroundings none of the best. I found him suffering with swollen joints, both ankles swollen, the right especially so. The left hip-joint was affected, the swelling being marked in the groin. The boy also complained of pains in the elbows and shoulders, but there was no perceptible swelling. Pulse, 140; temperature, 105 Fahr.; skin hot and dry; tongue moderately coated with light brown coat; respiration, normal. On inquiry of the parents, I found that four days previously the child, without prodromal symptoms, complained of chilliness, and that was followed by fever, and lameness and swelling of the ankles. The child had, according to their statement, been perfectly healthy up to that time. About a week prior to the attack he had fallen into the lake and had afterward played all day in his wet clothes. During the first three days of the child's sickness, his bowels had been constipated, so the mother said, and she had given some Ayer's pills that had produced two copious and offensive stools. On the fourth day, the child being no better, the parents had sent for me, as above stated.

I made a diagnosis of acute articular rheumatism, ordered a tepid sponge bath, for the sake of cleanliness, then wrapped the swollen joints in cotton-batting, gave salicylate of soda, 5 grains, every two hours, and an opiate to relieve pain. On the following day I found the patient apparently improved. Pulse 115; temperature 103; perspiring moderately. To relieve the sensitiveness of the swollen joints, I ordered them rubbed gently with composition chloroform liniment, which was occasionally varied with an ointment composed of ℥ij. of camphor chloral, ℥j. of lanolin. Under this treatment the pain and swelling subsided, with the exception of the right ankle which still remained much swollen and œdematous, the skin pitting on pressure; the pulse averaging 110 and the temperature ranging from 100 to 101. The color of the skin was almost natural over the swollen joints, being only slightly redder than normal.

On the fourth day of treatment, the patient complained of a dull pain in the region of the heart. Careful auscultation failed to detect any cardiac trouble or signs of pleuritic affection, but I ordered a mustard paste placed over the region of pain, which seemed to relieve it and the patient made no more complaints. The patient had occasional attacks of epistaxis. On the fifth day of treatment, the patient complained of nausea following the administration of the salicylate of soda. I lengthened the intervals between the doses from two to four hours and gave him tincture chlorid iron, 10 gtts. every four hours, alternately with the salicylate. On the fifth day of treatment found the patient more lively, wanting to sit up in bed. Every day during my attendance there had been a slight rise of temperature in the evening. On the sixth day, ordered the salicylate discontinued in the afternoon and in its place a 5-grain dose of antipyrin.

On the following day, the seventh of treatment and the eleventh from the inception of the disease, I was sent for in great haste with the message that the boy was worse and that there was a "hole in his leg." I went immediately and found the child in a moribund condition, unconscious, pulse 160 and almost imperceptible at the wrist. Respiration rapid, a forced long-drawn inspiration and short expiration; temperature in axilla 114. On turning back the bed-clothes found them saturated with a thin sanious pus, and about three inches above the right external malleolus an opening through which the pus had made its escape. The child died about twenty minutes after I arrived.

On inquiry found that the boy had taken his supper sitting in bed. During the night he became restless and slightly delirious; as the morning came on his fever increased, till at eleven o'clock he became unconscious with peculiar labored breathing. The parents, being stupid, allowed this to continue an hour before sending for me. During the whole course of the disease there was no skin eruption and up to the sixth day no signs to give a suspicion of suppuration around the swollen joints. A careful examination of the beginning of the treatment failed to find any bruise or injury of any kind that might give rise to suppuration and then cause pyæmia.

Dr. J. R. Brandt exhibited a specimen of

BILIARY CALCULUS.

This calculus was passed by Mrs. B. on May 28, 1884. She weighed 250 lbs., height 5 feet 10 inches, age 55 years.

Menstruation ceased at 48; she had never had jaundice, but had suffered from constipation all her life. This calculus weighed 4 ounces and 15 grains, measures 4 inches in circumference one way and 3¾ another way; it now weighs 3 ounces and 5 grains. It consists of cholesterin with dark bile pigmentation at one end; there are three facets at one end and one large one at the other. The lady was treated five months previously for a similar attack, which was diagnosed as malaria, at which time I think she passed the portion attached to the largest facet.

Gall-stones are not a frequent cause of intestinal obstruction, for in 1,152 cases collected by Leichtenstern there were only 41, and of these only 10 recovered. It is more common in females than in males, and in women above 50 years of age.

The points of interest are: The case might have been diagnosed as typhoid fever, as all the symptoms were there except the petechia and epistaxis, both of which are not constant. Then the size of the calculus, which is one of the largest, and perhaps the largest, (if we could have secured the other part) ever voided *per vias naturales.* Its being originally a gall-stone which remained lodged at the end of the duodenum for at least five months, and been bathed by the bile until it reached its present size. The recovery of the patient, where there is recorded a death rate of 25 per cent. Dr. Pye Smith was the first to make a diagnosis by rectal examination, when the calculus was at the end of the ilium by finding a rounded tumor of exceeding mobility.

